# Non-invasive ventilation alternating with high-flow nasal oxygen versus high-flow nasal oxygen alone after extubation in COPD patients: a post hoc analysis of a randomized controlled trial

**DOI:** 10.1186/s13613-021-00823-7

**Published:** 2021-02-09

**Authors:** Arnaud W. Thille, Rémi Coudroy, Mai-Anh Nay, Arnaud Gacouin, Maxens Decavèle, Romain Sonneville, François Beloncle, Christophe Girault, Laurence Dangers, Alexandre Lautrette, Quentin Levrat, Anahita Rouzé, Emmanuel Vivier, Jean-Baptiste Lascarrou, Jean-Damien Ricard, Keyvan Razazi, Guillaume Barberet, Christine Lebert, Stephan Ehrmann, Alexandre Massri, Jeremy Bourenne, Gael Pradel, Pierre Bailly, Nicolas Terzi, Jean Dellamonica, Guillaume Lacave, René Robert, Stéphanie Ragot, Jean-Pierre Frat, for the HIGH-WEAN Study Group, for the REVA Research Network, Florence Boissier, Maeva Rodriguez, Faustine Reynaud, Delphine Chatellier, Céline Deletage, Carole Guignon, Florent Joly, Anne Veinstein, Dalila Benzekri-Lefevre, Thierry Boulain, Grégoire Muller, Yves Le Tulzo, Jean-Marc Tadié, Adel Maamar, Suela Demiri, Julien Mayaux, Alexandre Demoule, Lila Bouadma, Claire Dupuis, Pierre Asfar, Marc Pierrot, Gaëtan Béduneau, Déborah Boyer, Benjamin Delmas, Bérénice Puech, Konstantinos Bachoumas, Edouard Soum, Séverin Cabasson, Marie-Anne Hoppe, Saad Nseir, Olivier Pouly, Gaël Bourdin, Sylvène Rosselli, Anthony Le Meur, Charlotte Garret, Maelle Martin, Guillaume Berquier, Abirami Thiagarajah, Guillaume Carteaux, Armand Mekontso-Dessap, Antoine Poidevin, Anne-Florence Dureau, Marie-Ange Azais, Gwenhaël Colin, Emmanuelle Mercier, Marlène Morisseau, Caroline Sabatier, Walter Picard, Marc Gainnier, Thi-My-Hue Nguyen, Gwenaël Prat, Carole Schwebel, Matthieu Buscot

**Affiliations:** 1 Centre Hospitalier Universitaire de Poitiers, Médecine Intensive Réanimation, Poitiers, France; 2 Centre d’Investigation Clinique 1402 ALIVE, Université de Poitiers, Poitiers, France; 3 Centre Hospitalier Régional d’Orléans, Médecine Intensive Réanimation, Orléans, France; 4 Service des Maladies Infectieuses et Réanimation Médicale, Centre Hospitalier Universitaire de Rennes, Hôpital Ponchaillou, Rennes, France; 5 Service de Pneumologie, Médecine Intensive et Réanimation (Département R3S), Groupe Hospitalier Universitaire APHP-Sorbonne Université, Hôpital Pitié-Salpêtrière, Paris, France; 6 Hôpital Bichat, Claude Bernard, Médecine Intensive Réanimation, AP-HP, Université de Paris, INSERM U1148, Paris, France; 7 Département de Médecine Intensive Réanimation, Centre Hospitalier Universitaire D’Angers, Université d’Angers, Angers, France; 8 Département de Réanimation Médicale, Centre Hospitalier Universitaire de Rouen, Hôpital Charles Nicolle, Institute for Research and Innovation in Biomedicine (IRIB), Normandie Université, UNIROUEN, EA3830-GRHV, Rouen, France; 9 Service de Réanimation Polyvalente, Centre Hospitalier Universitaire Félix Guyon, Saint Denis de la Réunion, France; 10 Service de Réanimation Médicale, Centre Hospitalier Universitaire de Clermont-Ferrand, Hôpital Gabriel Montpied, Clermont-Ferrand, France; 11 Service de Réanimation, Centre Hospitalier de La Rochelle, La Rochelle, France; 12 Centre de Réanimation, Centre Hospitalier Universitaire de Lille, 59000 Lille, France; 13 Réanimation Polyvalente, Centre Hospitalier Saint-Joseph Saint-Luc, Lyon, France; 14 Centre Hospitalier Universitaire de Nantes, Médecine Intensive Réanimation, Nantes, France; 15 Hôpital Louis Mourier, Réanimation Médico-Chirurgicale, AP-HP, INSERM, Université Paris Diderot, UMR IAME 1137, Sorbonne Paris Cité, Colombes, France; 16 Service de Réanimation Médicale, DHU A-TVB, AP-HP, Hôpitaux Universitaires Henri Mondor, Créteil, France; 17 Site Emile Muller, Service de Réanimation Médicale, Groupe Hospitalier Régional Mulhouse Sud Alsace, Mulhouse, France; 18 Service de Médecine Intensive Réanimation, Centre Hospitalier Départemental de Vendée, La Roche Sur Yon, France; 19 Centre Hospitalier Régional Universitaire de Tours, Médecine Intensive Réanimation, CIC 1415, Réseau CRICS-Trigger SEP, Centre d’étude des Pathologies Respiratoires, INSERM U1100, Université de Tours, Tours, France; 20 Centre Hospitalier de Pau, Service de Réanimation, Pau, France; 21 Centre Hospitalier Universitaire La Timone 2, Médecine Intensive Réanimation, Réanimation des Urgences, Aix-Marseille Université, Marseille, France; 22 Service de Réanimation, Centre Hospitalier Henri Mondor d’Aurillac, Aurillac, France; 23 Médecine Intensive Réanimation, Centre Hospitalier Universitaire de Brest, Brest, France; 24 Centre Hospitalier Universitaire Grenoble Alpes, Médecine Intensive Réanimation, INSERM, Université Grenoble-Alpes, U1042, HP2, Grenoble, France; 25 Centre Hospitalier Universitaire de Nice, Médecine Intensive Réanimation, Archet 1, UR2CA, Université Cote d’Azur, Nice, France; 26 Service de Réanimation Médico-Chirurgicale, Centre Hospitalier de Versailles, Le Chesnay, France; 27 Service de Médecine Intensive Réanimation, CHU de Poitiers, 2 rue la Milétrie, 86021 Poitiers Cedex, France

**Keywords:** Airway extubation, Weaning, Non-invasive ventilation, High-flow nasal oxygen, Chronic obstructive pulmonary disease

## Abstract

**Background:**

Several randomized clinical trials have shown that non-invasive ventilation (NIV) applied immediately after extubation may prevent reintubation in patients at high-risk of extubation failure. However, most of studies included patients with chronic respiratory disorders as well as patients without underlying respiratory disease. To date, no study has shown decreased risk of reintubation with prophylactic NIV after extubation among patients with chronic obstructive pulmonary disease (COPD). We hypothesized that prophylactic NIV after extubation may decrease the risk of reintubation in COPD patients as compared with high-flow nasal oxygen. We performed a post hoc subgroup analysis of COPD patients included in a multicenter, randomized, controlled trial comparing prophylactic use of NIV alternating with high-flow nasal oxygen versus high-flow nasal oxygen alone immediately after extubation.

**Results:**

Among the 651 patients included in the original study, 150 (23%) had underlying COPD including 86 patients treated with NIV alternating with high-flow nasal oxygen and 64 patients treated with high-flow nasal oxygen alone**.** The reintubation rate was 13% (11 out of 86 patients) with NIV and 27% (17 out of 64 patients) with high-flow nasal oxygen alone [difference, − 14% (95% CI − 27% to − 1%); *p* = 0.03]. Whereas reintubation rates were significantly lower with NIV than with high-flow nasal oxygen alone at 72 h and until ICU discharge, mortality in ICU did not differ between groups: 6% (5/86) with NIV vs. 9% (6/64) with high-flow nasal oxygen alone [difference − 4% (95% CI − 14% to 5%); *p* = 0.40].

**Conclusions:**

In COPD patients, prophylactic NIV alternating with high-flow nasal oxygen significantly decreased the risk of reintubation compared with high-flow nasal oxygen alone.

*Trial registration* The study was registered at http://www.clinicaltrials.gov with the trial registration number NCT03121482 (20 April 2017)

## Background

In intensive care units (ICUs), extubation failure rates leading to reintubation approximate 20–30% in patients with chronic respiratory disorders [1–7]. Several randomized clinical trials have shown that non-invasive ventilation (NIV) applied immediately after extubation may prevent reintubation in patients at high-risk of extubation failure [2, 7]. However, the patients in these studies considered at high-risk have been heterogeneous and included patients with chronic respiratory disorders as well as patients without underlying respiratory disease. Whether prophylactic application of NIV after extubation reduces reintubation rate in patients with chronic obstructive pulmonary disease (COPD) has been poorly assessed. Previous clinical trials have shown that NIV could be particularly effective to prevent post-extubation respiratory failure in patients with chronic respiratory disorders, but again, these studies did not specifically focus on COPD patients and showed beneficial effects of NIV only in hypercapnic patients [3, 4]. To date, only one small-scale trial has compared NIV with standard oxygen exclusively in COPD patients, and this study failed to highlight the beneficial effects of NIV [8]. Conversely, high-flow nasal oxygen could be as effective as NIV in preventing reintubation in patients at high-risk [9]. Therefore, we performed a post hoc subgroup analysis of a recent large-scale randomized controlled trial showing that prophylactic NIV alternating with high-flow nasal oxygen significantly reduced the risk of reintubation as compared to high-flow nasal oxygen alone in a heterogeneous population of patients at high-risk of extubation failure [7].

We hypothesized that prophylactic NIV alternating with high-flow nasal oxygen after extubation may be particularly effective in COPD patients and may decrease the risk of reintubation in this subgroup of patients as compared with high-flow nasal oxygen alone.

## Methods

### Study design and patients

The present study is a post hoc analysis of a multicenter, randomized, controlled trial comparing prophylactic use of NIV alternating with high-flow nasal oxygen versus high-flow nasal oxygen alone immediately after extubation in 641 patients at high-risk of reintubation in ICUs [7]. The present analysis focused on the subset population of COPD patients. Underlying COPD could be either documented by spirometry or highly suspected by physician team. Patients who were considered as having underlying COPD without previous spirometry needed to have been intubated for acute hypercapnic respiratory failure and for no other reason, and to have a history of smoking with dynamic hyperinflation during mechanical ventilation and/or emphysema on chest X-ray or scanner. Patients with long-term treatment with NIV or continuous positive airway pressure at home for sleep obstructive apnea syndrome were excluded. The original study was approved by the central ethics committee (Ethics Committee Ouest III, Poitiers, France) with registration number 2016-A01078-43. Written informed consent was obtained from all patients or next of kin before inclusion. According to French law, this post hoc analysis did not require further ethics approval.

### Treatment groups

All patients were extubated after successful spontaneous breathing trial and received prophylactic NIV alternating with high-flow nasal oxygen or high-flow nasal oxygen alone during the first 48 h following extubation. In the two groups, treatment could be continued beyond the first 48 h following extubation until complete recovery of respiratory status. Patients assigned to the control group were continuously treated by high-flow nasal oxygen alone with a flow of 50 L/min and fraction of inspired oxygen (FiO_2_) adjusted to obtain adequate oxygenation, with a pulse oximetry (SpO_2_) at least 92%. Patients assigned to the interventional group were treated by NIV alternating with high-flow nasal oxygen. Non-invasive ventilation was initiated immediately after extubation with a first session of at least 4 h and minimal duration of at least 12 h a day during the 48 h following extubation. NIV was carried out with an ICU ventilator with NIV mode or dedicated bi-level ventilator, in pressure-support mode with a minimal pressure-support level of 5 cm H_2_O targeting a tidal volume around 6–8 ml/kg of predicted body weight, a positive end-expiratory pressure level between 5 and 10 cmH_2_O and a FiO_2_ adjusted to obtain adequate oxygenation (SpO_2_ ≥ 92%). Between non-invasive ventilation sessions, high-flow nasal oxygen was delivered as in the control group.

### Outcomes

The main outcome was reintubation rates within the 7 days following extubation according to oxygenation strategy. Secondary outcomes included post-extubation respiratory failure within the 7 days following extubation, reintubation rates at 48 h, 72 h and up until ICU discharge, and mortality in ICU.

Severe respiratory failure leading to reintubation was defined by the presence of at least two criteria among the following: respiratory rate > 35 breaths per minute, clinical signs of respiratory distress, respiratory acidosis defined as pH < 7.25 units and PaCO_2_ > 45 mm Hg, hypoxemia defined as FiO_2_ ≥ 80% to maintain SpO_2_ ≥ 92% or a PaO_2_/FiO_2_ ≤ 100 mm Hg.

An episode of post-extubation respiratory failure was defined by the presence of at least two criteria among the following: respiratory rate > 25 breaths per minute, clinical signs of respiratory distress, respiratory acidosis defined as pH < 7.35 units and PaCO_2_ > 45 mm Hg, and hypoxemia defined as FiO_2_ ≥ 50% to maintain SpO_2_ ≥ 92% or a PaO_2_/FiO_2_ ≤ 150 mm Hg.

### Statistical analysis

Continuous variables were expressed as mean ± standard deviation (SD) or median [interquartile range, 25–75th percentiles], and qualitative variables were expressed as number and percentage. Patients’ characteristics were compared between the NIV group and the high-flow nasal oxygen group by means of the *χ*^2^ tests or Fisher exact test for categorical variables and Student’s *t*-test or Wilcoxon rank-sum test for continuous variables as appropriate. Primary and secondary outcomes were compared between the two groups by means of the *χ*^2^ test. Kaplan–Meier curves were plotted to assess the time from extubation to reintubation and were compared by means of the log-rank test at day 7. The results were presented as odds ratio (OR) with 95% confidence interval (95 CI). A two-tailed *p* value of less than 0.05 was considered statistically significant. We used SAS software, version 9.4 (SAS Institute), for all the analyses.

## Results

Among the 651 patients extubated in the 30 participating ICUs, 150 (23%) had underlying COPD including 102 patients (68%) with COPD confirmed by spirometry. Median forced expiratory volume during the first second (FEV1) was 58% [interquartile range, 42–71%] expressed in percentage of predicted value according to sex and age. Among the 86 patients in whom FEV1 was available, 44% (38 of 86 patients) had severe COPD (stage 3 or 4 according to the Gold classification, i.e., FEV1 < 50% of predicted value).

In overall population, the main reason for intubation at admission was acute respiratory failure in 102 patients (68%), with as main diagnosis bacterial pneumonia in 35% of cases (*n* = 36), severe acute exacerbation without identified diagnosis in 33% (*n* = 34), viral pneumonia in 14% (*n* = 14), cardiogenic pulmonary edema in 11% (*n* = 11), and another reason in 7% (pulmonary embolism, pneumothorax, pleural effusion, hemoptysis, airway obstruction, and aspiration). Among the 102 patients intubated for acute respiratory failure, 36% (*n* = 37) had been intubated prior to ICU admission.

Of the 150 COPD patients, weaning was considered as simple in 102 patients (68%), difficult in 44 (29%), and prolonged in 4 (3%). At time of extubation, 53 patients (35%) had hypercapnia (PaCO_2_ > 45 mm Hg). After extubation, 86 patients were treated with prophylactic NIV alternating with high-flow nasal oxygen and 64 with high-flow nasal oxygen alone. The characteristics of the patients at inclusion were similar in the two groups aside from a higher proportion of patients with ineffective cough in the NIV group (Table 1).

**Table 1:** Baseline patient characteristics according to the oxygenation strategy used after extubation

	High-flow nasal oxygen alone (*n* = 64)	Non-invasive ventilation (*n* = 86)	*p* value
Characteristics of the patients at admission			
Age, years	66 ± 9	66 ± 9	0.900
Male sex, *n* (%)	48 (75%)	61 (71%)	0.580
Body mass index (BMI), kg/m^2^	27 ± 6	27 ± 7	0.875
Obesity (BMI ≥ 30 kg/m^2^), *n* (%)	14 (22%)	25 (29%)	0.326
COPD confirmed by spirometry, *n* (%)	43 (67%)	53 (62%)	0.483
FEV1, % of predicted value	58 ± 18	57 ± 17	0.834
Underlying chronic cardiac disease, *n* (%)	14 (22%)	28 (33%)	0.149
Ischemic heart disease	9 (14%)	15 (17%)	0.577
Atrial fibrillation	4 (6%)	9 (10%)	0.364
Left ventricular dysfunction	5 (8%)	7 (8%)	0.942
SAPS II at admission, points	53 ± 17	52 ± 18	0.874
Main reason for intubation, *n* (%)			
Acute respiratory failure	43 (67%)	59 (69%)	0.099
Coma	6 (9%)	10 (12%)	
Shock	9 (14%)	3 (3%)	
Cardiac arrest	4 (6%)	4 (5%)	
Surgery	2 (3%)	9 (10%)	
Other reasons	0 (0%)	1 (1%)	
Characteristics of the patients on the day of extubation			
SOFA score, points	4.0 ± 2.5	3.8 ± 2.0	0.639
Median duration of mechanical ventilation, days	5.5 [3.0–10.5]	6.5 [3.0–13.0]	0.504
Difficult or prolonged weaning#, *n* (%)	20 (33%)	28 (32%)	0.865
Ineffective cough, *n*/*n* total (%)	7/59 (12%)	26/82 (32%)	*0.006*
Abundant secretions, *n*/*n* total (%)	31/60 (52%)	37/83 (45%)	0.402
Administration of steroids before extubation, *n* (%)	12 (19%)	20 (23%)	0.505
Ventilator settings before the spontaneous breathing trial (SBT)			
Pressure support ventilation, *n* (%)	58 (91%)	75 (87%)	0.514
Pressure support level, cm H_2_O	9.6 ± 2.5	9.6 ± 3.0	0.997
Positive end-expiratory pressure, cm H_2_O	5.6 ± 1.7	6.0 ± 1.7	0.225
Tidal volume, ml/kg	7.8 ± 2.2	7.9 ± 2.4	0.687
Respiratory rate, breaths/min	23 ± 7	22 ± 6	0.767
FiO_2_, %	34 ± 8	37 ± 13	0.228
PaO_2_/FiO_2_, mm Hg	248 ± 70	247 ± 87	0.916
pH, units	7.45 ± 0.05	7.44 ± 0.05	0.529
PaCO_2_, mm Hg	44 ± 9	43 ± 8	0.373
PaCO_2_ > 45 mm Hg, *n* (%)	26 (41%)	30 (35%)	0.542
Characteristics of the spontaneous breathing trial (SBT)			
Type of SBT, *n* (%)			0.807
T-piece, *n* (%)	37 (58%)	48 (56%)	–
Low level of pressure-support ventilation, *n* (%)	27 (42%)	38 (44%)	–
Median duration of the SBT, min	60 [30–62]	60 [30–60]	0.432
Respiratory rate at the end of SBT, breaths/min	24 ± 7	24 ± 6	0.782
PaO_2_ at the end of SBT, mm Hg (*n* = 109)	78 ± 18	79 ± 22	0.735
pH at the end of SBT, units (*n* = 109)	7.45 ± 0.05	7.46 ± 0.05	0.586
PaCO_2_ at the end of SBT, mm Hg (*n* = 109)	44 ± 10	42 ± 9	0.398
Hypercapnia at time of extubation^‡^, *n* (%)	22 (34%)	31 (36%)	0.832

Italic means that there is a significant difference (*p* < 0.05)

Continuous variables are given in mean ± standard deviation or median [interquartile range, IQR 25–75th percentiles] according to their distribution

*COPD* chronic obstructive pulmonary disease, *FEV1* forced expiratory volume during the first second (expressed in % of predicted value according to sex and age), *SAPS* Simplified Acute Physiology Score, *SOFA* Sepsis-Related Organ Failure Assessment, *SBT* Spontaneous Breathing Trial

^#^Difficult or prolonged weaning refer to patient who failed the first spontaneous breathing trial and were not extubated the day of the first trial

^‡^Hypercapnia (defined as PaCO_2_ > 45 mm Hg) was assessed according to the PaCO_2_ level measured at the end of the spontaneous breathing trial (109 patients) or at the end of or under mechanical ventilation before the trial if this latter was not measured (41 patients)

Ventilator settings using NIV were the following: pressure-support level of 7.9 ± 2.4 cm H_2_O, PEEP level of 5.2 ± 1.3 cm H_2_O, and FiO_2_ of 0.34 ± 0.9, resulting in a tidal volume of 8.8 ± 3.6 ml per kilogram of predicted body weight. Patients treated with high-flow nasal oxygen alone received a gas flow rate of 50 ± 3 L/min with FiO_2_ of 0.42 ± 0.13. NIV was delivered for a median of 14 h [interquartile range, 10–16] within the first 24 h following extubation and for 23 h [interquartile range, 14–29] within the first 48 h. In the NIV group, NIV was continued beyond the first 48 h for incomplete recovery of respiratory status in 28 patients (33%) whereas in the high-flow nasal oxygen group, high-flow nasal oxygen was continued in 26 patients (41%) (difference − 8%; 95 CI, − 23% to 7%; *p* = 0.309).

### Outcomes

**Fig. 1:**
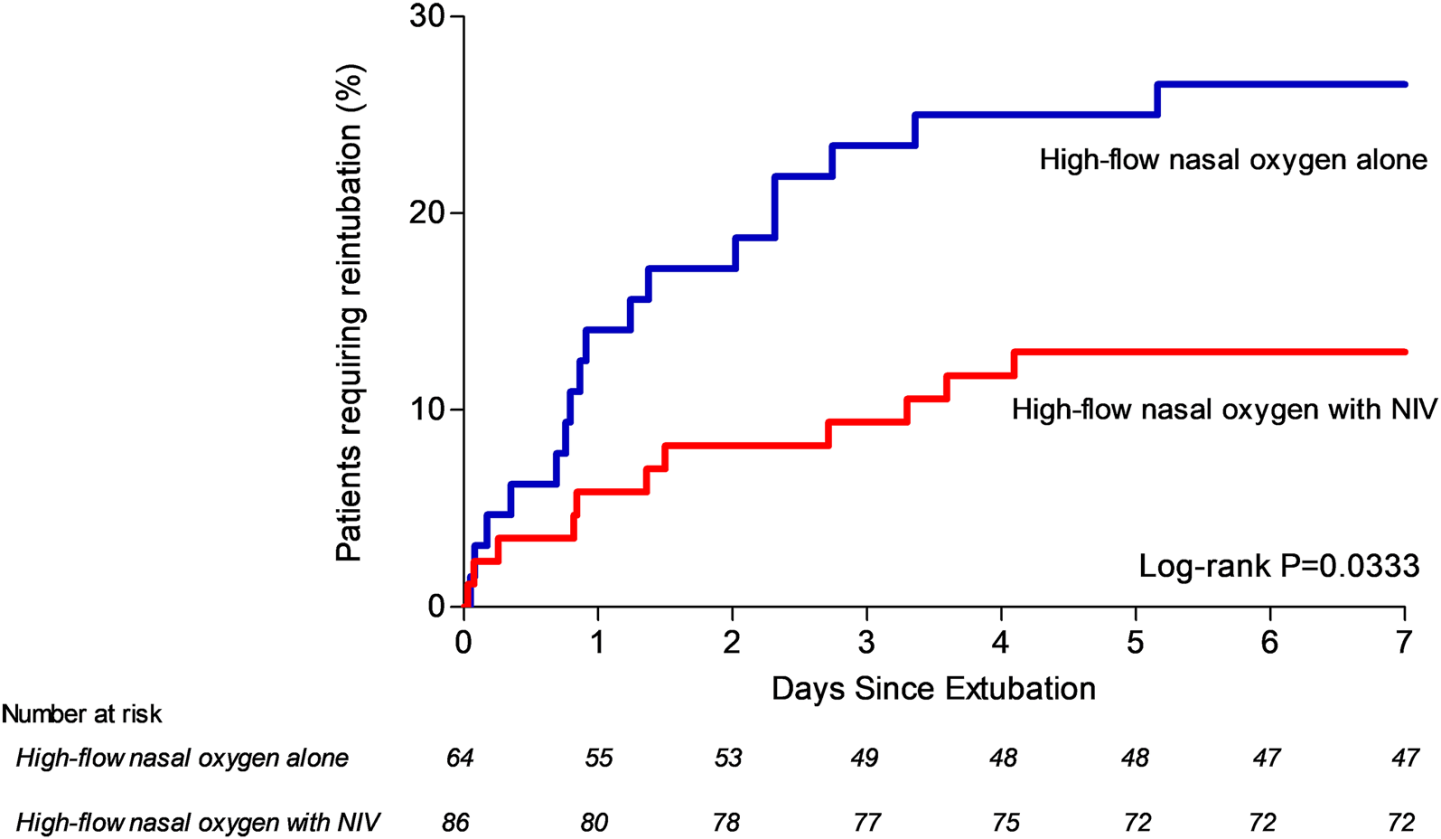
Kaplan–Meier analysis of time from extubation to reintubation according to oxygenation strategy. Reintubation rates were significantly lower in patients treated with non-invasive ventilation (red bars) than in those treated with high-flow nasal oxygen alone (blue bars). The reintubation rate within the 7 days following extubation was 13% (11 out of 86 patients) with NIV and 27% (17 out of 64 patients) with high-flow nasal oxygen alone [difference, − 14% (95% CI − 27% to − 1%); *p* = 0.033 using log-rank test]

The reintubation rate at day 7 was 13% (11 out of 86 patients) with NIV and 27% (17 out of 64 patients) with high-flow nasal oxygen alone [difference − 14% (95% CI − 27% to − 1%); *p* = 0.03] (Fig. 1). Reintubation rates were significantly lower with NIV than with high-flow nasal oxygen alone at 72 h and until ICU discharge (Table 2).

**Table 2:** Primary and secondary outcomes

	Non-invasive ventilation (*n* = 86)	High-flow nasal oxygen alone (*n* = 64)	Absolute difference % (95% CI)	*p* value
Primary outcome				
Reintubation at day 7, *n* (%)	11 (13%)	17 (27%)	− 14 (− 27 to − 1)	*0.032*
Secondary outcomes				
Post-extubation respiratory failure, *n* (%)	22 (26%)	23 (36%)	− 10 (− 25 to 4)	0.171
Use of NIV to treat post-extubation respiratory failure, *n* (%)	19/22 (86%)	6/23 (26%)	60 (32 to 76)	*< 0.001*
Reintubation at day 7 among patients with post-extubation respiratory failure, *n* (%)	10/22 (45%)	14/23 (61%)	− 15 (− 40 to 13)	0.300
Reintubation at 48 h, *n* (%)	7 (8%)	11 (17%)	− 9 (− 21 to 2)	0.092
Reintubation at 72 h, *n* (%)	8 (9%)	15 (23%)	− 14 (− 27 to − 2)	*0.018*
Reintubation in ICU, *n* (%)	11 (13%)	18 (28%)	− 15 (− 28 to − 2)	*0.019*
Length of stay in ICU, median (IQR), days	13 [8–21]	12 [8-19]	–	0.908
Length of stay in hospital, median (IQR), days	26 [14–47]	23 [16–32]	–	0.406
Mortality in ICU, *n* (%)	5 (6%)	6 (9%)	− 4 (− 14 to 5)	0.408
Mortality in hospital, *n* (%)	15 (17%)	8 (12%)	5 (− 7 to 16)	0.406

Italic means that there is a significant difference (*p* < 0.05)

*NIV* non-invasive ventilation, *CI* confidence interval, *ICU* intensive care unit, *IQR* interquartile range (25–75th percentiles)

The proportion of patients with post-extubation respiratory failure did not significantly differ between groups. The proportion of patients who received NIV to treat post-extubation respiratory failure was 86% (19 of 22 patients) in the NIV group and 26% (6 of 23 patients) in the high-flow oxygen group [difference 60% (95% CI 32–76%); *p* < 0.001]. However, the proportion of patients reintubated among those who experienced post-extubation respiratory failure did not significantly differ between the 2 groups.

Mortality in ICU did not differ between groups: 6% (5/86) with NIV vs. 9% (6/64) with high-flow nasal oxygen alone [difference − 4% (95% CI − 14% to 5%); *p* = 0.40].

## Discussion

In this post hoc analysis of a randomized controlled trial focusing on COPD patients, prophylactic use of NIV alternating with high-flow nasal oxygen immediately after extubation significantly decreased the rate of reintubation as compared with high-flow nasal oxygen alone.

This trial is the largest one reporting the effects of prophylactic NIV after extubation in COPD patients. Previously, only one small-scale trial comparing NIV with standard oxygen exclusively in COPD patients failed to highlight the beneficial effects of NIV [8]. Another clinical trial showing decreased risk of post-extubation respiratory failure in patients with chronic respiratory disorders treated with NIV included a majority of COPD patients (74 of 106 patients) [4]. However, the risk of reintubation was not significantly lower with NIV than with standard oxygen and all patients had hypercapnia at time of extubation. Here, we report for the first time decreased risk of reintubation in COPD patients treated with prophylactic NIV after extubation.

### Limitations

The main limitation of this study is the post hoc nature of the analysis. However, characteristics of the patients were similar between the 2 groups and the reintubation rates observed are very close to reintubation rates reported in previous studies (around 10% with NIV and between 20 and 30% with standard oxygen) reinforcing external validity [5, 7–9]. The only imbalance between groups was a higher proportion of patients with ineffective cough in the NIV group, situation associated with an increased risk of extubation failure [10, 11]. Despite this unfavorable imbalance, prophylactic NIV was associated with a decreased risk of reintubation. This is in keeping with a previous study reporting that NIV may avoid reintubation in patients with weak cough as compared with standard oxygen [12].

Another major limitation is that more than one-third of patients had suspected but not confirmed COPD using spirometry. These patients were admitted for their first acute exacerbation of COPD or never underwent pulmonary function tests planned after hospital discharge, as is often the case. Indeed, several studies have reported the difficulty for follow-up of these patients and their reluctance to come back for pulmonary explorations after hospital discharge [13, 14]. However, all these patients were admitted for acute hypercapnic respiratory failure and had common risk factors of COPD. Consequently, it is likely that the majority of patients with suspected obstructive spirometric pattern actually had underlying COPD.

Although NIV was the one additional treatment in the interventional group, the decreased risk of reintubation observed in the NIV group might be due to the combination of NIV with high-flow nasal oxygen between NIV sessions. Although the beneficial effects of NIV on oxygenation, alveolar ventilation, and work of breathing are well-demonstrated [15, 16], continuation of high-flow nasal oxygen between NIV sessions may provide further clinical improvement by decreasing work of breathing [17, 18]. Whereas prophylactic NIV could be strongly recommended in patients at high-risk of extubation failure and especially in COPD patients, whether NIV alternating with high-flow nasal oxygen is a better oxygenation strategy than NIV alternating with standard oxygen is a question that requires further investigation.

Lastly, the proportion of patients who experienced post-extubation respiratory failure as well as the proportion of patients reintubated among those with respiratory failure did not significantly differ. However, both rates were reduced, resulting in a significant decreased risk of reintubation with NIV as compared with high-flow nasal oxygen alone. Patients receiving prophylactic NIV were more likely to be treated with NIV in case of post-extubation respiratory failure than those receiving high-flow nasal oxygen alone. As a result, NIV may prevent post-extubation respiratory failure and subsequently avoid reintubation among patients with respiratory failure. Even though the most recent international clinical practice guidelines suggest that NIV should not be used in the treatment of patients with established post-extubation respiratory failure [19], NIV as rescue therapy may avoid reintubation in a number of cases, especially in patients with chronic obstructive pulmonary disease [3–5, 20].

### Clinical implications

Several studies have suggested that prophylactic NIV after extubation may be particularly effective in hypercapnic patients [3, 4, 7]. However, less than 40% of patients included in our study had hypercapnia, and the majority of patients had moderate underlying COPD with a mean forced expiratory volume during the first second (FEV1) above 50% of predicted value. It should be emphasized that patients had not necessarily been intubated for acute exacerbation of COPD and that nearly one-third of them had been intubated for another reason than acute respiratory failure (cardiac arrest, shock, coma, or surgery). Prophylactic NIV may decrease the risk of reintubation even in patients with mild or moderate COPD and regardless of the level of PCO_2_ before extubation, and thereby, NIV should be applied in all COPD patients to prevent extubation failure in the ICU.

## Conclusion

Prophylactic use of NIV alternating with high-flow nasal oxygen immediately after extubation of COPD patients was associated with decreased risk of reintubation compared with high-flow nasal oxygen alone.

## Data Availability

The datasets used and/or analyzed during the current study are available from the corresponding author on reasonable request.
